# Functional connectivity density aberrance in type 2 diabetes mellitus with and without mild cognitive impairment

**DOI:** 10.3389/fneur.2024.1418714

**Published:** 2024-06-10

**Authors:** Limin Ge, Zidong Cao, Zhizhong Sun, Xiaomei Yue, Yawen Rao, Kui Zhao, Wenbin Qiu, Yifan Li, Weiye Lu, Shijun Qiu

**Affiliations:** ^1^First Clinical Medical College, Guangzhou University of Chinese Medicine, Guangzhou, China; ^2^Department of Endocrinology, Shenzhen Traditional Chinese Medicine Hospital, Shenzhen, China; ^3^Department of Radiology, The First Affiliated Hospital of Guangzhou University of Chinese Medicine, Guangzhou, China; ^4^State Key Laboratory of Traditional Chinese Medicine Syndrome, Guangzhou, China

**Keywords:** type 2 diabetes mellitus, mild cognitive impairment, functional connectivity density, functional connectivity, functional magnetic resonance imaging purpose

## Abstract

**Purpose:**

The objective of this study was to investigate alterations in functional connectivity density (FCD) mapping and their impact on functional connectivity (FC) among individuals diagnosed with Type 2 diabetes mellitus (T2DM) across different cognitive states. Moreover, the study sought to explore the potential association between aberrant FCD/FC patterns and clinical or cognitive variables.

**Methods:**

A total of 211 participants were recruited for this study, consisting of 75 healthy controls (HCs), 89 T2DM patients with normal cognitive function (DMCN), and 47 T2DM patients with mild cognitive impairment (DMCI). The study employed FCD analysis to pinpoint brain regions exhibiting significant FCD alterations. Subsequently, these regions showing abnormal FCD served as seeds for FC analysis. Exploratory partial correlations were conducted to explore the relationship between clinical biochemical indicators, neuropsychological test scores, and altered FCD or FC.

**Results:**

The FCD analysis revealed an increased trend in global FCD (gFCD), local FCD (lFCD), and long-range FCD (lrFCD) within the bilateral supramarginal gyrus (SMG) among individuals with DMCN. Additionally, significant lFCD alterations were observed in the right inferior frontal gyrus and left precuneus when comparing DMCN to HCs and DMCI.

**Conclusion:**

When comparing individuals with T2DM and healthy controls (HCs), it was revealed that DMCN exhibited significant improvements in FCD. This suggests that the brain may employ specific compensatory mechanisms to maintain normal cognitive function at this stage. Our findings provide a novel perspective on the neural mechanisms involved in cognitive decline associated with T2DM.

## Introduction

The prevalence of Type 2 diabetes mellitus (T2DM) is characterized by disrupted glucose metabolism, typically in the context of insulin resistance and metabolic syndrome (1). T2DM poses a significant threat to human health, ranking closely behind cancer and cardiovascular disease. There is an escalating global prevalence of T2DM across all geographical regions. According to the 10th diabetes atlas from the International Diabetes Federation (IDF), it is projected that there will be approximately 700 million individuals with diabetes by 2045, with about 90–95% attributed to T2DM (2). Increasing evidence from clinical and fundamental research suggests that individuals with T2DM are susceptible to mild cognitive impairment (MCI) and ultimately the development of dementia (3–5). MCI represents a condition between age-appropriate cognition and dementia, which is considered as the primary state of vulnerability for Alzheimer’s disease (AD) and has been associated with increased mortality rates in individuals with diabetes (6–8). Therefore, imperative research on the pathophysiological mechanisms underlying MCI in individuals with T2DM is necessary to facilitate timely intervention and enhance prognosis.

The utilization of resting-state fMRI (rs-fMRI) has gained prevalence in cognitive impairment research, facilitating the investigation and comprehension of the underlying neural mechanisms in the early stages of cognitive disorders. Previous studies have consistently demonstrated that specific brain regions exhibit abnormal neuronal activity in individuals with T2DM across various cognitive states (9). In a separate study, T2DM patients with MCI (DMCI) displayed significantly more extensive alterations in regional homogeneity and functional brain networks compared to T2DM patients with normal cognitive function (DMCN) (10). Furthermore, an exhaustive analysis of intranetwork and internetwork functional connectivity (FC) also revealed a broader impairment among patients with DMCI (11). These findings suggest the presence of compensatory mechanisms during the development of cognitive decline in T2DM. However, limited attention has been given to modifications in functional connectivity density (FCD) mapping and their impact on FC among patients with varying cognitive states in T2DM.

FCD quantifies the number of functional connections between a specific voxel and other voxels in the brain across multiple dimensions. The FCD value of a voxel is directly proportional to its effective FC, indicating that higher FCD values are crucial in maintaining brain function. In subsequent FC analyses, FCD methodology enables the network’s characteristics to identify and pinpoint node regions, rather than relying on *a priori* selection of specific seed regions (12, 13). It encompasses three categories: local FCD (lFCD), global FCD (gFCD), and long-range FCD (lrFCD), which are determined by neighboring voxel connections (14). The gFCD indicates the extent of functional coupling throughout the brain, while the lFCD reflects local alterations, and the lrFCD demonstrates integration of non-contiguous voxels. Various metrics can be used to define different aspects of a functional hub (13). Employing a multiscale assessment can enhance disease identification accuracy (15, 16).

Our study aimed to comprehensively assess abnormalities in the functional connectivity of DMCN, DMCI, and HC using a combination of FCD analyses and seed-based FC analyses. This approach was chosen because solely relying on FCD analyses does not provide insights into specific regions exhibiting abnormal connectivity with altered FCD regions (17). In addition, we conducted correlation analyses to explore the relationship between aberrant FCD/FC and clinical/cognitive factors. We hypothesized that patients with DMCN and DMCI would display distinct variations in FCD and FC patterns, which could contribute to cognitive decline and help differentiate various cognitive states of T2DM.

## Materials and methods

### Participants

T2DM patients, consisting of 47 DMCI and 89 DMCN patients, were recruited from October 2021 to April 2023 at the First Affiliated Hospital of Guangzhou University of Chinese Medicine for this study. Additionally, 75 healthy controls (HCs) were chosen from the community following medical examinations. Criteria for inclusion involved being right-handed and aged between 30 and 70 years. The diagnostic criteria for T2DM were based on the guidelines proposed by the American Diabetes Association (1). Patients with T2DM were receiving stable treatment, which included following a prescribed diet, oral medication, and/or insulin. In the case of the DMCI group, inclusion criteria were: (1) complaining of memory decline; (2) a mini-mental state exam (MMSE) score > 24 and a Montreal Cognitive Assessment (MoCA) score < 26; and (3) preserved general functional abilities. For all participants, exclusion criteria included: (1) any other neurological or psychiatric disorders that could cause cognitive impairment; (2) any other systemic disease; and (3) contraindications for MRI scanning. Participants in the DMCN group and the HCs group were regarded to have normal cognitive function (MMSE >24 and MoCA ≥26). Ethical approval for this study was obtained from the Medical Research Ethics Committee of our hospital, and all participants provided written informed consent prior to participating in the study protocol.

### Clinical and neuropsychological measurements

We documented the height, weight, body mass index (BMI), and arterial blood pressure of all participants. Additionally, laboratory examinations were performed on T2DM patients, encompassing glycosylated hemoglobin (HbA1c), fasting blood glucose (FBG), fasting insulin (FINS), total cholesterol (TC), triglyceride (TG), and low-density lipoprotein (LDL).

All participants underwent a battery of neuropsychological tests, including the MoCA (18), MMSE (19), Auditory Verbal Learning Test (AVLT) (20), Grooved Pegboard Test (GPT) (21), Digit Span Test (DST) (22), Clock-Drawing Test (CDT) (23), Digit Symbol Substitution Test (DSST) (24), and Trail-Making Test Part A (TMT-A) (25). Each subject completed the full battery of tests in a predetermined sequence, requiring approximately 1 h per individual.

### MRI acquisition

MRI images were acquired using a 3.0 T MR scanner (MAGNETOM Prisma, Siemens, Germany) equipped with a 64-channel head coil. T2-weighted and T2-FLAIR sequences were routinely obtained to rule out organic brain lesions. For the rs-fMRI images, the echo planar sequence was employed with the following parameters: field of view (FOV) = 244 mm × 244 mm, echo time (TE) = 30 ms, repetition time (TR) = 500 ms, thickness = 3.5 mm, voxel size = 3.5 × 3.5 × 3.5 mm, slices = 35, flip angle = 60°, and a total of 960 volumes were acquired. The parameters for the 3D T1WI sequence are as follows: inversion time (TI) = 1,100 ms, TR = 2,530 ms, TE = 2.98 ms, FOV = 256 mm × 256 mm, thickness = 1 mm, voxel size = 1 × 1 × 1 mm, flip angle = 7°, and a total of 192 sagittal slices. All participants were instructed to close their eyes and remain conscious during the scan.

### Preprocessing of resting-state fMRI

The rs-fMRI data underwent preprocessing using the DPABI^1^ software package and was processed in MATLAB 2022b (26). Initially, 10 time points were excluded to ensure magnetic field stability. The remaining 950 time points underwent head motion correction realignment. For images with a shorter echo time, performing slice timing during preprocessing was deemed unnecessary (27). Participants with head motion greater than 2 mm and/or a translation greater than 2° of rotation were excluded from this study. To achieve more accurate spatial normalization, the DARTEL method was employed to guide the rs-fMRI registration. In order to regress the head motion effects from realignment, the Friston 24-parameter head motion model was used, which included 6 head motion parameters, 6 head motion parameters from the preceding time point, and the 12 corresponding squared values (28). White matter, cerebrospinal fluid, and linear drift signals were also regressed as nuisance variables. Finally, filtering the data within a 0.01–0.1 Hz range reduced noise.

### FCD mapping

We utilized the Neuroscience Information Toolbox (NIT, version 1.3) (29) to evaluate the FCD mapping for each subject. Specifically, we conducted Pearson correlation analyses to assess voxel-wise functional correlations. Voxels demonstrating a correlation coefficient exceeding 0.6 were considered significantly associated (13). The global FCD value for each voxel was determined by quantifying its significant functional connections with other voxels in the gray matter (12, 13). Similarly, the local FCD value of a voxel was calculated by identifying the number of spatially connected voxels exhibiting a significant correlation. Subtracting lFCD from gFCD allowed for the acquisition of the lrFCD map for each participant. Moreover, the FCD maps underwent smoothing using a Gaussian kernel with a full-width at half maximum (FWHM) of 6 × 6 × 6 mm, and to standardize the maps, they were divided by the mean FCD value.

### FC analysis

We conducted additional FC analyses to explore the direct functional connections between brain regions showing alterations in the FCD maps. The coordinates of the peak point in the FCD analysis results were selected as the center of spherical regions of interest (ROIs) with a 6-millimeter radius. Subsequently, a Pearson correlation analysis was carried out between the ROI and the voxels within the gray matter mask of the entire brain to generate FC maps. Afterward, Fisher’s r-z transformation was applied to all FC maps to ensure normality. Additionally, the FC maps underwent smoothing with a FWHM of 6 × 6 × 6 mm.

### Statistical analyses

The statistical analysis was conducted using the Statistical Package for the Social Sciences (IBM, SPSS, version 26). The normality of demographic, clinical, and neuropsychological data was assessed using the Shapiro–Wilk test. Disparities in the data between the HC, DMCN, and DMCI groups were assessed using analysis of variance (ANOVA), the χ^2^-test, and the Kruskal-Wallis test. Furthermore, additional post-hoc comparisons were conducted using the Bonferroni correction, with the significance level set at *p* < 0.05.

A one-way ANOVA was performed utilizing the DPABI software to analyze the differences in FCD and FC among three groups, with age, gender, education level, and head movement parameters as covariates. Additionally, we applied the false discovery rate (FDR) correction at the voxel level, setting the significance threshold at *p* < 0.05, and stipulating a minimum cluster size of 100 voxels. Subsequently, we computed the mean FCD or FC values for each cluster displaying significant differences across the three groups and compared these values between each group pair using a two-sample *t*-test and the Bonferroni correction.

We utilized partial correlations in T2DM patients to examine the connections between clinical biochemical indicators, scores of neuropsychological tests, and altered FCD or FC, while adjusting for gender, age, and education level. Additionally, we set the statistical significance level at *p* < 0.05.

## Results

### Demographic, clinical, and cognitive characteristics

Upon examination, no significant statistical differences in gender, age, and BMI were observed across the three groups. The DMCN group notably displayed higher levels of education and blood pressure. There were no statistical discrepancies found in clinical biochemical indicators between the DMCN and DMCI groups. Furthermore, the DMCI group exhibited lower performance on various tests, including MoCA, MMSE, AVLT, GPT (R), DST (inverse), CDT, DSST, and TMT-A (Table 1).

**Table 1:** Demographic and clinical data of all participants.

	HCs (*n* = 75)	DMCN (*n* = 89)	DMCI (*n* = 47)	Statistics	*p-*value
Age (years)	47.71 ± 8.77	48.91 ± 7.88	50.45 ± 7.49	*F* = 1.651	0.194
Gender (male/female)	30/45	49/40	26/21	χ^2^ = 4.437	0.109
BMI (kg/m^2^)	23.51 ± 2.52	23.82 ± 3.19	24.63 ± 3.26	*F* = 2.082	0.127
Education (years)	9(8, 12)	12(9, 15)	9(7, 14)	*z* = 18.703	**<0.001** ^**ac**^
SBP (mmHg)	122.03 ± 14.50	128.56 ± 15.53	127.48 ± 18.37	*F* = 3.398	**0.035** ^**a**^
DBP (mmHg)	80.90 ± 9.29	85.67 ± 11.75	82.80 ± 11.29	*F* = 3.678	**0.027** ^**a**^
HbA1c (%)	N/A	7.40(6.50, 9.50)	8.4 (7.20, 9.50)	*z* = 2.889	0.089
FBG (mmol/L)	N/A	7.54(6.21, 9.06)	7.88(6.66, 10.32)	*z* = 0.809	0.369
FINS (μIU/mL)	N/A	8.13(5.22, 13.91)	10.51(5.11, 16.15)	*z* = 0.676	0.411
HOMR-IR	N/A	2.90(1.72, 5.09)	3.61(2.11, 6.34)	*z* = 1.060	0.249
TG (mmol/L)	N/A	1.60(1.00, 2.27)	1.84(1.24, 2.22)	*z* = 1.138	0.286
TC (mmol/L)	N/A	4.81(4.16, 5.53)	4.73(4.15, 5.12)	*z* = 1.247	0.264
LDL (mmol/L)	N/A	3.16(2.61, 3.68)	3.18(2.51, 3.57)	*z* = 0.462	0.497
MoCA	27(26, 28.5)	28(27, 28)	23(22, 25)	*z* = 112.717	**<0.001** ^**bc**^
MMSE	29(27,30)	29(28, 29)	28(26, 28)	*z* = 16.193	**<0.001** ^**bc**^
AVLT (immediate)	23.23 ± 4.99	23.79 ± 4.51	21.02 ± 5.37	*F* = 5.093	**0.007** ^**bc**^
AVLT (5 min)	9(8, 10)	10(9, 11)	8(6, 9.5)	*z* = 27.538	**<0.001** ^**abc**^
AVLT-delay	9(8, 10)	10(8, 11)	7(5, 9)	*z* = 25.813	**<0.001** ^**bc**^
AVLT-recall	12(11, 12)	12(12, 12)	11.5(8, 12)	*z* = 10.883	**0.004** ^**c**^
GPT (R)	68(59.74, 78.5)	71(64, 79)	74.5(66, 87)	*z* = 8.865	**0.012** ^**b**^
GPT (L)	76(66.5, 86)	78(71, 87)	84.5(69, 97)	*z* = 5.698	0.058
DST (forward)	8(6, 8)	8(6.5, 8)	7(5, 8)	*z* = 1.973	0.373
DST (inverse)	4(4, 5)	5(4, 5)	4(3, 5)	*z* = 7.852	**0.020** ^**c**^
CDT	3(3, 4)	4(4, 4)	3(3, 4)	*z* = 19.089	**<0.001** ^**ac**^
DSST	43(33.5, 50.5)	45.5(38, 55)	33.5(28, 43)	*z* = 18.305	**<0.001** ^**bc**^
TMT-A	48.5(37, 63)	42 (35, 51.5)	57(41, 73.67)	*z* = 14.573	**<0.001** ^**c**^

Data are presented as N, median (Q1, Q3), and mean ± SD. N/A, not applicable. BMI, body mass index; SBP, systolic blood pressure; DBP, diastolic blood pressure; HbA1c, hemoglobin A1c; FBG, fasting blood glucose; FINS, fasting insulin; HOMA-IR, homeostatic model assessment of insulin resistance; TG, triglyceride; TC, total cholesterol; LDL, low-density lipoprotein; MoCA, Montreal cognitive assessment; MMSE, mini-mental state examination; AVLT, auditory verbal learning test; GPT, grooved pegboard test; DST, Digit Span Test; CDT, Clock-Drawing Test; DSST, digit symbol substitution test; TMT-A, trail making test-A. ^a^*Post hoc* analyses showed a significant group difference between HC and DMCN. ^b^*Post hoc* analyses showed a significant group difference between HC and DMCI. ^c^*Post hoc* analyses showed a significant group difference between DMCN and DMCI. The meaning of the bolded values represents reaching the significant threshold (*p* < 0.05) with statistical significance.

### FCD analyses

**Figure 1:**
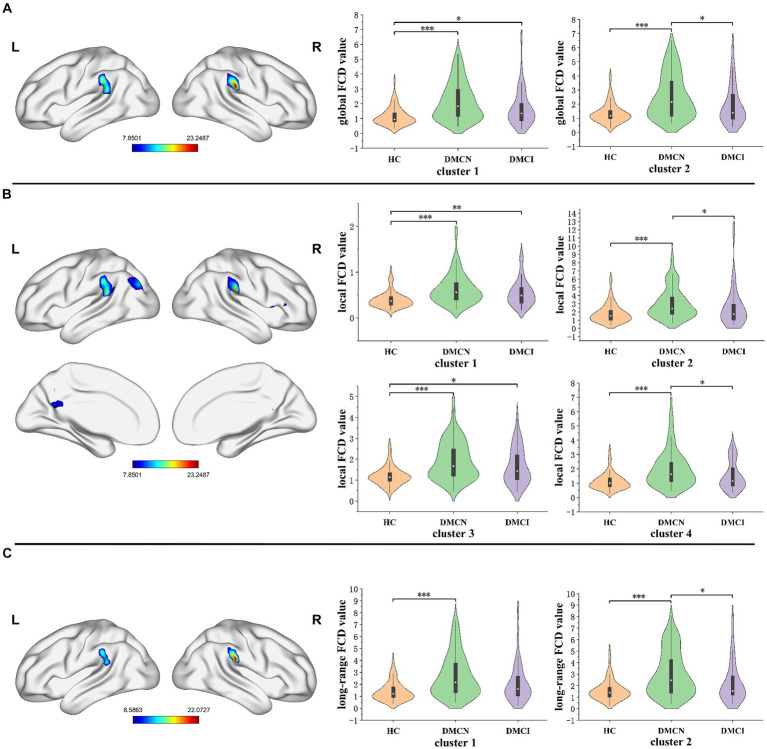
The distribution of brain regions with statistical differences in the FCD mapping for DMCN, DMCI, and HCs. **(A)** The results of significant differences in global FCD across three groups. **(B)** The results of significant differences in local FCD across three groups. **(C)** The results of significant differences in long-range FCD across three groups. FDR corrected, *p* < 0.05, the cluster size >100 voxels. FCD, functional connectivity density. *p < 0.05, **p < 0.01, ***p < 0.005.

The statistical analysis of FCD revealed significant variations in gFCD, lFCD, and lrFCD values across the three groups. The ANOVA analysis for gFCD demonstrated statistically significant changes in the bilateral supramarginal gyrus (SMG). Post-hoc results revealed an increasing trend in patients with DMCN compared to HCs and DMCI. The results of ANOVA analysis for lFCD indicated a significant statistical disparity in several brain regions, including the right orbital inferior frontal gyrus, the right opercular inferior frontal gyrus (IFGorb_R/IFGoperc_T), the left precuneus (PCUN_L), the left superior temporal gyrus, the left supramarginal gyrus, the left middle occipital gyrus, the left angular gyrus (STG_L/SMG_L/MOG_L/ANG_L), and the right supramarginal gyrus (SMG_R). Additionally, the three groups showed significant differences in lrFCD in the bilateral SMG. Furthermore, in the post-hoc analysis, patients with DMCN exhibited significantly higher values of lFCD and lrFCD in the altered brain regions compared to HCs and DMCI (Table 2; Figure 1).

**Table 2:** Global, local, and long-range FCD alterations across three groups.

FCD	Cluster index	Cluster size (voxels)	Brain regions (aal)	Peak MNI coordinate (x, y, z)	Peak intensity (*t*-value)
Global FCD	1	171	SupraMarginal_L	−60, −33, 33	17.6681
	2	145	SupraMarginal_R	57, −27, 24	23.2487
Local FCD	1	173	Frontal_Inf_Orb_R// Frontal_Inf_Oper_R	48, 18, −12	14.5797
	2	290	Precuneus_L	−3, −57, 21	12.4588
	3	527	Temporal_Sup_L// SupraMarginal_L// Occipital_Mid_L// Angular_L	−60, −36, 21	18.4162
	4	175	SupraMarginal_R	54, −27, 24	19.203
Long-range FCD	1	102	SupraMarginal_L	−60, −33, 33	15.7847
	2	110	SupraMarginal_R	57, −27, 24	22.0727

Using the anatomical automatic labeling (AAL) template. Cluster size > 100 voxels in ANOVA analysis, *P* < 0.05, FDR corrected. FCD, functional connectivity density; MNI, Montreal Neurological Institute; X, Y, Z, coordinates of primary peak locations in the MNI space.

### FC analyses

**Figure 2:**
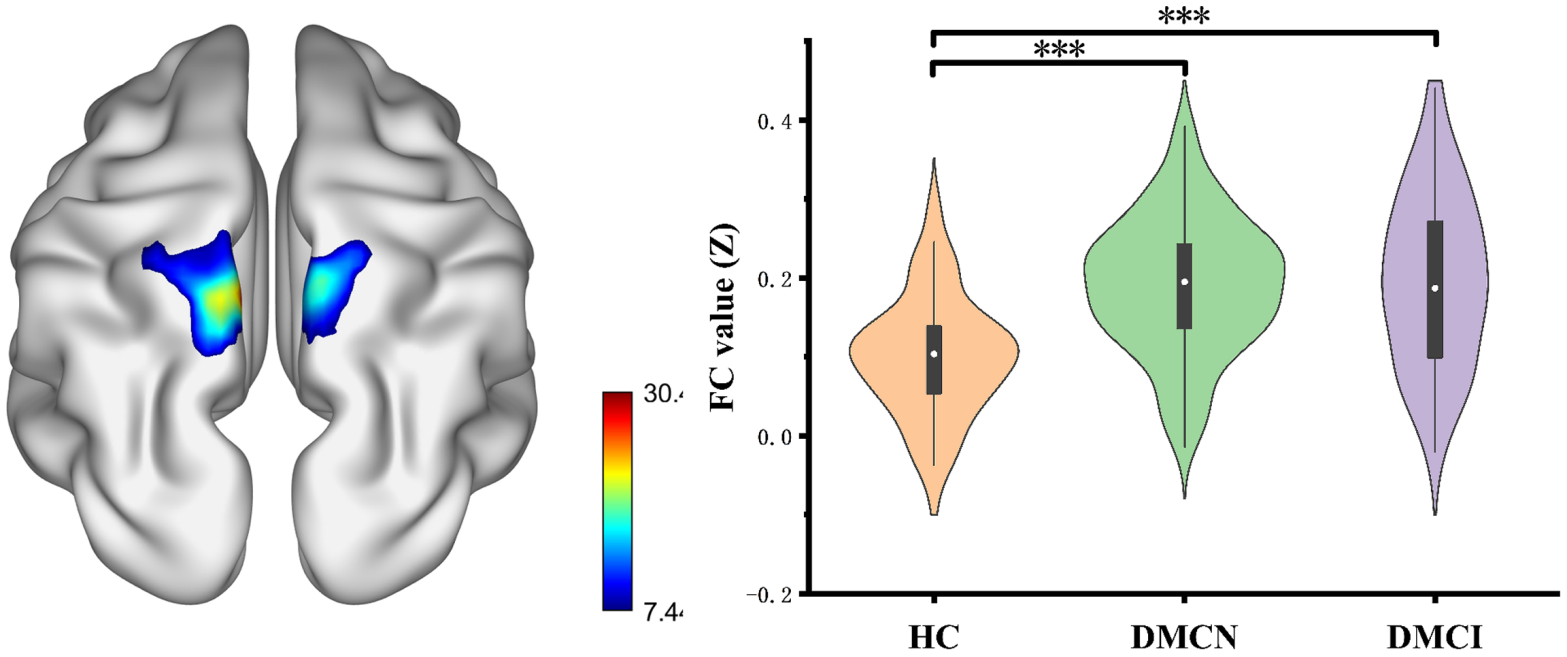
Results of the FC analyses with the SMG.R as a seed. Show results of the significant changes among groups (one-way ANOCA, FDR corrected, *p* < 0.05, the cluster size >100 voxels). FC, functional connectivity. ***p < 0.005.

After conducting FDR correction, no statistical significance was found for the SMG_L seed at coordinates (*x* = −60, *y* = −33, *z* = −33) across the three groups. Conversely, the ANOVA analysis indicated significant differences in the bilateral precuneus for the SMG_L seed at coordinates (*X* = 57, *Y* = −27, *Z* = 24). Notably, the functional connectivity (FC) value of the bilateral precuneus was significantly higher in individuals with DMCN and DMCI in comparison to HCs (Table 3; Figure 2).

**Table 3:** Regions with changed resting-state FC based on seed-based analyses across three groups.

Seed (MNI-sphere)	Cluster index	Cluster size (voxels)	Brain regions (aal)	Peak MNI coordinate (x, y, z)	Peak intensity (*t*-value)
ROI1 (*x* = −60, *y* = −33, *z* = 33)	NA				
ROI2 (*x* = 57, *y* = −27, *z* = 24)	1	698	Precuneus_L// Precuneus_R	−9, −48, 54	30.4255

Using the anatomical automatic labeling (AAL) template. Cluster size > 100 voxels in ANOVA analysis, *p* < 0.05, FDR corrected. FC, functional connectivity; ROI, region of interest; MNI, Montreal Neurological Institute; X, Y, Z, coordinates of primary peak locations in the MNI space.

### Correlation analyses

**Figure 3:**
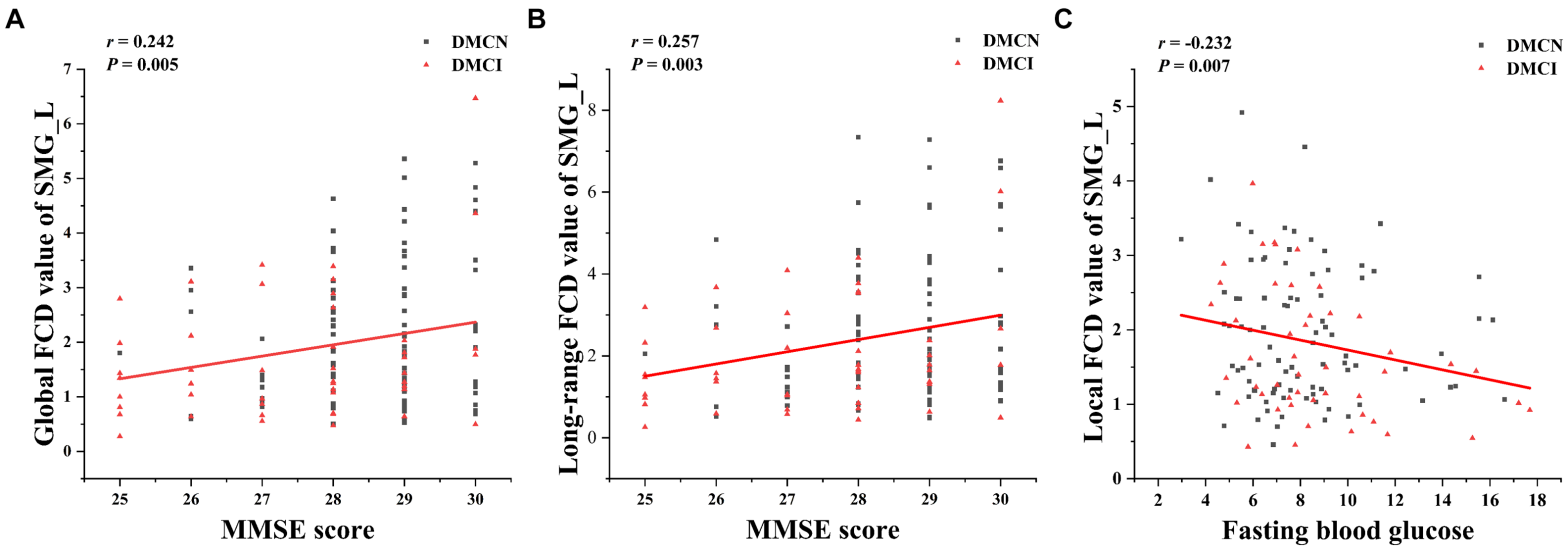
The correction analysis. **(A)** Global FCD value of SMG_L was positively correlated with MMSE score (*p* = 0.005, *r* = 0.242). **(B)** Long-range FCD value of SMG_L was positively correlated with MMSE score (*p* = 0.003, *r* = 0.257). **(C)** Local FCD value of SMG_L was negatively correlated with fasting blood glucose (*p* = 0.047, *r* = −0.360).

Partial correlation analysis in the DMCN and DMCI groups showed a prominent positive association between the aberrant gFCD and lrFCD values in the SMG_L and the MMSE score. Additionally, the modified lFCD in SMG.L was found to be inversely correlated with FBG (Figure 3).

## Discussion

The primary objective of the study was to investigate alterations in functional connectivity density (FCD) maps and related functional connectivity (FC) in individuals with type 2 diabetes mellitus (T2DM), both with and without mild cognitive impairment (MCI). Our research findings indicated that patients with T2DM exhibited abnormalities in FCD, primarily affecting the bilateral supramarginal gyrus (SMG), right inferior frontal gyrus (IFG), and left precuneus. The analysis of FC supported the results from the FCD analyses, highlighting the bilateral precuneus as a key region for altered FC. Furthermore, additional correlation studies among individuals with T2DM revealed a significant association between aberrant FCD/FC and clinical/cognitive factors, which holds paramount importance. Overall, these results are crucial for enhancing our understanding of the neural mechanisms underlying diverse cognitive states associated with T2DM.

The significant variations in the gFCD, lFCD, and lrFCD between the three groups were found in the bilateral supramarginal gyrus. The inferior parietal lobe, which includes the supramarginal gyrus and the angular gyrus, is part of the default mode network (DMN), a significant contributor to cognitive processing (30, 31). Furthermore, the supramarginal gyrus plays a vital role in language processing, and its deficiencies may be linked to decreased language processing resulting from persistent dysglycemia (32, 33). Although it is widely acknowledged that individuals with T2DM experience mild neuronal impairment, they nonetheless can maintain regular clinical and cognitive functioning due to sufficient functional compensation. In this study, we observed that the FCD value of the bilateral supramarginal gyrus in T2DM was significantly higher than that of HCs, indicating that the bilateral supramarginal gyrus formed a more effective functional connection with other regions. Previous studies on topological alterations of the brain functional network showed that DMCI patients had considerably higher global and local efficiency, as well as multiple nodal centralities, compared to HCs. However, the topological properties of the entire brain network did not display any significant abnormalities among DMCN patients (34). The difference may be attributed to methodological and/or clinical factors. Furthermore, global topological measurements’ sensitivity is insufficient to identify minimal changes in the initial stages of the disease. In a comparison of two T2DM subgroups (DMCN and DMCI), the FCD value of DMCN markedly increased, aligning with prior research on Alzheimer’s disease. These findings may suggest that functional compensation is observed in all individuals with T2DM, but it is insufficient in patients with DMCI to compensate for neurological deterioration, resulting in mild cognitive impairment. Our hypothesis posited that the absence of sufficient compensatory mechanisms in individuals indicated additional neural function impairment.

In the realm of lFCD, patients diagnosed with T2DM exhibited a heightened change in the right inferior frontal gyrus and left precuneus, in comparison with HCs. The right inferior frontal gyrus, identified as a vital region for executive control (35), plays a crucial role in numerous advanced cognitive functions like working memory and attention (36–38). Previous studies have shown abnormal gFCD changes in the right IFG in patients with T2DM, suggesting that this region is vulnerable to T2DM-related brain damage (39). Additionally, the outcome was congruous with the results of cognitive tests. Earlier research has proposed that the precuneus functions as the central hub of the DMN and is associated with increased levels of amyloid deposition during the early stages of AD (40). Alterations in the functional connectivity of the precuneus may be employed as an effective biomarker for predicting the onset of AD (41). Our hypothesis is that this particular increase in elevation might be an indication of functional compensation, potentially serving as a distinctive biomarker to differentiate between DMCN and DMCI.

In our study, using the right supramarginal gyrus as the seed, we observed significant changes in FC in the bilateral precuneus region in patients with DMCN and DMCI. Notably, these observations are consistent with FCD analyses, and the precuneus is an important component of the default mode network (42). It is well established that damage to the precuneus and inferior parietal lobule results in memory impairment (41). Previous studies on the default mode network have established that the precuneus functional defect serves as a reliable neuroimaging biomarker for cognitive impairment related to T2DM (43). Our findings provide further confirmation that T2DM patients exhibit compensatory connectivity in the precuneus. Additionally, when utilizing the left supramarginal gyrus as the seed, we did not observe any significant alteration. This could be attributed to our application of a more rigorous multiple comparison correction method. Moving forward, we plan to increase the sample size and employ a more refined approach.

In patients with T2DM, the study observed a positive correlation between the gFCD and lrFCD of the left supramarginal gyrus and the MMSE score, that is to say, as the functional connectivity density increases, the cognitive function scores of T2DM patients also increase correspondingly. These findings provided additional support for the association between enhanced functional compensation and the enhancement of general cognitive function. Furthermore, the study revealed a negative correlation between the altered lFCD in SMG_L and FBG levels. FBG levels in patients with T2DM are linked to a heightened risk of cognitive impairment, and there is a persistent association between blood sugar levels and cognitive decline. Our study provides additional evidence, from a neuroimaging standpoint, of the potential impact of FBG on the cognitive function of individuals with T2DM by influencing FC (44, 45).

It is important to acknowledge that our study has several limitations. The statistical results may have been somewhat influenced by the relatively small sample size, limiting the inferences that can be drawn. However, due to the ongoing updates to our database, additional participants will be included in the future. Furthermore, there was no complete alignment in educational levels across the three groups, potentially affecting the outcomes. To address this issue, we included gender, age, education, and head movement as covariates in FCD/FC statistical analyses. Moreover, the majority of enrolled patients had varying disease durations and were on different medications, making it difficult to determine the impact of disease duration and medication on certain outcomes. Therefore, future research should focus on mitigating these confounding factors. Lastly, our analysis only compared variations between different groups at a single time point and did not investigate the progression from normal to impaired cognition in individuals with T2DM. To comprehensively evaluate changes in brain imaging in T2DM patients at various cognitive states, additional longitudinal studies are necessary to validate our findings.

## Conclusion

The study investigated aberrant FCD and seed-based FC in patients with T2DM across different cognitive states. Altered brain regions were found to be distributed throughout the default mode network. When comparing HCs and DMCI, it was observed that individuals with DMCI had a significant increase in FCD, suggesting that the brain may employ compensatory mechanisms to maintain normal cognitive function during this stage. These findings enhance our understanding of altered brain activity in patients with T2DM and its impact on cognitive performance. Furthermore, these abnormalities may provide potential indicators, based on functional neuroimaging, that could be utilized for early detection of cognitive decline.

## Data availability statement

The raw data supporting the conclusions of this article will be made available by the authors, without undue reservation.

## Ethics statement

The studies involving humans were approved by the Medical Research Ethics Committee of Guangzhou University of Chinese Medicine. The studies were conducted in accordance with the local legislation and institutional requirements. The participants provided their written informed consent to participate in this study.

## Author contributions

LG: Writing – original draft. ZC: Writing – original draft. ZS: Writing – original draft. XY: Writing – original draft. YR: Writing – original draft. KZ: Writing – original draft. WQ: Writing – original draft. YL: Writing – original draft. WL: Writing – original draft. SQ: Writing – review & editing.

## Footnotes

^1^http://rfmri.org/dpabi/
